# Supplementary material for: Using Qualitative Methods to Explore Farrier-Related Barriers to Successful Farriery Interventions for Equine Welfare in India
Source: Animals (Basel). 2019 May 18;9(5):252. doi: 10.3390/ani9050252 (PMC6562430; doi:10.3390/ani9050252)
Supplement: Supplementary file 1 [file animals-09-00252-s001.pdf]

Informed Consent Form for Farriery Research Study Project, 2016

I am Dr.Dinesh S Mohite working for Brooke (India). I am doing research on farriery and its challenges which is common in this region. This form is to ask your consent to participate in this study. Before you decide, you can talk to anyone you feel comfortable with about the research. If you have any questions later, you can ask them of me.

1. Purpose of the study

We believe that you can help us to understand the difficulties, challenges you face during this profession in this context. We also want to understand what enables you to do your job well. We also want to know how our trainings/mentoring useful for you and how much you learnt from those. We also want to learn how your practices has been changed or not and reasons behind the same. The aim of the study is to gather information in order to improve our farriery programme.

2. Procedure

This study will be carried out through group discussion between 6 to 8 local farriers. This group discussion will be facilitated by a researcher and recorded by a separate recorder on paper as well as voice recorder. Facilitator will ask you open ended questions related to your job and give you time to share your knowledge. We will not ask you to share your personal information like family, your net income, however we will ask your sources of income other than farriery job whosever applicable. You do not have to share any information that you are not comfortable sharing. The duration of discussion will be maximum two hours. The discussion will hold in a place where most of the participant can come and join easily. It will also carry out as per the participant's suitable time, preferably off working times. The discussion will not carry out on participant's prayer time if any. The discussion will carry out in calm and quite place to avoid distractions. The recorded information will be downloaded on researchers desktop and password protected in Brooke India's head office.

3. Harms & Benefits

The discussion will be held nearby your working area so that once you finish your work you can join easily. Because the group discussion will take time, there is a possibility that you may not take as many client on that particular day, but equids and equid owners may wait for your services and take them on different day. Some of you may travel a long distances on that particular day to participate in discussion. There may not be direct benefits to you, but this research will help us to improve our Farriery programme which may help you in your work.

4. Compensation, Costs and Reimbursement

You will not be provided any incentive or compensation towards your work, but we will reimburse your travel expenses (to and fro) and receipt of the same will be taken for our record purpose. We will also provide food to you on the discussion day.

5. Withdrawal or Termination from study

You do not have to take part in this study if you do not wish to do so, and choosing to participate will not affect your job or job-related evaluation in any way. You may stop participating in the discussion at any time that you wish without your job being affected. In few circumstances, we may feel you are not required for the discussion then we may terminate your participation without your consent. We

will give you an opportunity at the end of the discussion to review your remarks, and you can ask to modify or remove portions of those, if you do not agree with our notes or if we did not understand you correctly.

6. Anonymity and confidentiality

You will not be identified by name on the tape and we will replace names if any by codes for analysis and publication. The generated data will be stored in Brooke India's head office on researchers desktop and protected by password. The information recorded is confidential, and no one else except researcher in Brooke India and UK will have access. The tapes will be destroyed after five years of completion of study. The data/findings will be shared with Brooke India and UK staff, scientific community (publications/conferences) for the purpose of strategic intervention and wider dissemination respectively.

7. Findings

The results and recommendations of the study will be shared with you by March, 2017 once the final study report approved by Brooke UK. This will be done through any forum organized by our Brooke India district unit staff in your area.

8. Alternatives to Participation

You may refuse or suggest any other participant to participate in this study. We will make you understand about why you have been selected to participant in this study.

9. Conflicts of interest

Brooke India or researcher(s) or their staff does not have a financial or professional or social or personal relationship with other people or organizations that could inappropriately influence or bias the content of this study.

10. Contact information

Mr. Martand Singh, Manager, Meerut, (U.P.), Brooke India; Mobile No.+91-945739**** Mr.Shivkumar Tomar, Manager, Muzaffarnagar district (U.P.), Brooke India; Mobile No. +91-941059****, Dr. Dinesh S Mohite, Team Leader Research, Brooke India; +91-882643****

11. Verification of understanding and consent

You might have understood the project and its implications. Could you tell me the aim, potential harms and benefits of this project in your own words? If you have any questions, you can ask them now. Do you know that you do not have to take part in this study if you do not wish to? You can say No if you wish to? Do you know that I have given the contact details of the person who can give you more information about the study?

I have read the foregoing information, or it has been read to me. I have had the opportunity to ask questions about it and any questions I have been asked have been answered to my satisfaction. I consent voluntarily to be a participant in this study

Name of Participant____________________________________

Signature of Participant __________________

Date ________________

If illiterate

(The witness must be impartial, such as an adult who is not a member of the research team and who is not a family member of the participant)

I have witnessed the accurate reading of the consent form to the potential participant, and the individual has had the opportunity to ask questions. I confirm that the individual has given consent freely.

Name of witness_________________________

Signature of witness __________________

Date ____________

Thumb print of participant

Statement by the researcher/person taking consent

I confirm that the participant was given an opportunity to ask questions about the study, and all the questions asked by the participant have been answered correctly and to the best of my ability I confirm that the individual has not been forced into giving consent, and the consent has been given freely and voluntarily.

A copy of this informed consent form has been provided to the participant.

Name of Researcher/person taking the consent________________________________

Signature of Researcher /person taking the consent____________________

Date ________________
